# Risk of systemic reactions during pediatric allergen-specific immunotherapy: clinical predictors and safety outcomes

**DOI:** 10.55730/1300-0144.6347

**Published:** 2026-05-11

**Authors:** Figen ÇELEBİ ÇELİK, Alp KAZANCIOĞLU, Soner GÜNDER, Melike OCAK, Berna UZUNOĞLU, Canan Şule KARKINER, Özlem SANCAKLI, Ayfer TUNCER, Özge UYSAL SOYER, Bülent Enis ŞEKEREL, Demet CAN, Ümit Murat ŞAHİNER

**Affiliations:** 1 Division of Pediatric Allergy and Immunology, Department of Pediatrics, Dr. Behcet Uz Pediatric Diseases and Surgery Training and Research Hospital, İzmir Faculty of Medicine, University of Health Sciences, İzmir, Turkiye; 2 Division of Pediatric Allergy, Department of Pediatrics, Faculty of Medicine, Hacettepe University, Ankara, Turkiye

**Keywords:** Asthma, pediatric, pollen, subcutaneous immunotherapy, systemic reactions

## Abstract

**Background/aim:**

This study aimed to evaluate the frequency, timing, severity, and clinical predictors of systemic reactions in children receiving subcutaneous allergen-specific immunotherapy (SCIT) with house dust mite (HDM) and grass pollen extracts under real-world clinical conditions.

**Materials and methods:**

This multicenter retrospective study included 623 children with moderate-to-severe allergic rhinitis, with or without asthma, who received SCIT between 2006 and 2023 at two tertiary pediatric allergy centers. Clinical characteristics, laboratory findings, immunotherapy protocols, and systemic reactions (SRs) were recorded and graded according to the updated 2024 World Allergy Organization (WAO) systemic reaction scale.

**Results:**

SCIT was well tolerated. Systemic reactions occurred in 10.7%, corresponding to 0.27% per injection. Most SRs were mild-to-moderate (WAO grades 1–2), and approximately three-quarters occurred during the maintenance phase. A progressive decline in SR incidence was observed throughout the treatment period. In the pollen SCIT subgroup, asthma was the only independent predictor of SRs (OR, 2.35; 95% CI 1.13–4.88; p = 0.022). No life-threatening reactions or fatalities were observed.

**Conclusion:**

Pediatric SCIT with pollen and HDM extracts demonstrated a favorable safety profile, with infrequent and predominantly nonsevere systemic reactions. The association between asthma and SRs in pollen SCIT highlights the importance of optimal asthma control throughout immunotherapy.

## 1. Introduction

Allergen-specific immunotherapy remains the only disease-modifying treatment available for immunoglobulin E (IgE)-mediated allergic diseases such as allergic rhinitis and asthma. Numerous clinical trials and long-term follow-up studies have shown that subcutaneous allergen-specific immunotherapy (SCIT) reduces symptoms, limits medication requirements, and provides durable clinical benefits [1,2]. It is generally well tolerated; however, adverse events may occur, most of which are local, whereas systemic reactions (SRs) occur less frequently and may be severe or even life-threatening [3,4]. Extensive surveillance efforts have reassured clinicians that SCIT is safe when administered appropriately. Data from North American surveillance programs covering tens of millions of injections suggest that fatal outcomes are extremely rare, with an estimated rate of approximately one death per nine million injection visits [3]. Nonetheless, SRs that necessitate treatment with epinephrine still arise in clinical practice, emphasizing the need for vigilance during therapy [4]. Given this potential risk, identifying clinical and treatment-related predictors of SRs is of considerable clinical importance.

Previous studies have reported several potential risk factors for SRs, including uncontrolled asthma, multiple sensitizations, dosing errors, starting a new extract bottle, and use of expedited administration schedules [5–7]. Children are a particularly relevant group in this context. They represent a substantial proportion of patients treated with immunotherapy; however, most safety data are derived from adult populations. Pediatric reports suggest that SRs may occur more frequently in children, but available studies are limited in size and scope [6,7]. Despite these reports, predictors of SRs in children receiving SCIT have not been fully established, and pediatric data remain limited compared with those available for adults.

House dust mites and grass pollen are among the most common aeroallergens associated with allergic rhinitis and asthma in children. Although SCIT with these allergens is widely prescribed, multicenter real-world data on its safety profile, including systemic reaction rates, timing, and associated risk factors in children, remain limited.

Therefore, the current multicenter study was designed to evaluate the frequency and timing of SRs in children receiving SCIT with house dust mite and grass pollen extracts and to investigate potential clinical and treatment-related predictors of such reactions. By addressing this knowledge gap, evidence may be provided to help optimize risk stratification and support the safe use of SCIT in the pediatric population.

## 2. Materials and methods

### 2.1. Patient selection and data collection

This retrospective, multicenter study included pediatric patients with AR and/or asthma who underwent SCIT at two tertiary care allergy clinics in Türkiye (Hacettepe University and Dr. Behcet Uz Pediatric Diseases and Surgery Training and Research Hospital). Allergic rhinitis was diagnosed according to the Allergic Rhinitis and Its Impact on Asthma (ARIA) guidelines, and asthma was diagnosed according to the Global Initiative for Asthma (GINA) recommendations [8,9]. Medical data from children aged 5 to 18 years who received SCIT with house dust mite or grass pollen extracts between January 2006 and December 2023 were systematically reviewed. Polysensitized patients with sensitizations to allergens other than house dust mites and grass pollen were excluded from the study.

Demographic characteristics, clinical diagnosis, serum total IgE levels, peripheral eosinophil counts and percentages, details of the immunotherapy protocol, number of injections, and the occurrence, severity, and timing of SRs were recorded. Ethical approval was obtained before data collection from the Institutional Ethics Committee of Dr. Behcet Uz Pediatric Diseases and Surgery Training and Research Hospital (approval no. GOA-238), and informed consent was obtained from the patients and/or their parents in accordance with the Declaration of Helsinki.

### 2.2. Subcutaneous allergen-specific immunotherapy

Standard depot allergen extracts commercially available in Türkiye were used. ALK-Abellò (Madrid, Spain) and Allergopharma (Reinbek, Germany) preparations were used for grass pollen SCIT, whereas Novo-Helisen (Allergopharma, Reinbek, Germany) and Phostal (Stallergenes, Antony Cedex, France) preparations were used for house dust mite SCIT. All patients received conventional SCIT administered according to a conventional schedule, which was initiated either before or after the relevant allergen season.

The build-up schedules were implemented in accordance with the manufacturers’ recommendations. Increases were administered weekly during the build-up phase. Monthly injections were administered during the maintenance phase.

All injections were administered by physicians or experienced allergy nurses in outpatient clinics. Before each injection, patients were assessed for current symptoms and their response to the previous dose. Injections were given subcutaneously into the upper arm, with patients observed for at least 30 min following each dose. Dose adjustments or treatment postponements were made in accordance with national guideline recommendations in cases of intercurrent infection, uncontrolled asthma, or recent systemic or large local reactions. Systemic reactions were carefully documented.

### 2.3. Classification and grading of systemic reactions

Systemic reactions were graded according to the updated World Allergy Organization (WAO) Subcutaneous Immunotherapy Systemic Reaction Grading System (2024) [10]. This revised grading system distinguishes five severity levels from grade 1 (mild, single-organ system involvement) to grade 5 (fatal anaphylaxis) and introduces further subclassifications for grade 1 symptoms to increase consistency and comparability across studies. Grade 1 refers to mild symptoms affecting a single organ system and is now divided into three subgroups: skin symptoms such as generalized pruritus or urticaria, upper airway symptoms such as sneezing, nasal congestion, or rhinorrhea, and ocular symptoms such as redness, itching, or tearing. Grade 2 describes moderate reactions involving more than one organ system that are not life-threatening, such as urticaria accompanied by mild wheezing or gastrointestinal symptoms. Grade 3 indicates severe reactions, typically with lower airway involvement such as bronchospasm, marked shortness of breath, or laryngeal edema, but without cardiovascular collapse. Grade 4 represents life-threatening anaphylaxis with hypotension, hypoxemia, or loss of consciousness requiring urgent intervention. Grade 5 is defined as a fatal outcome.

### 2.4. Statistical analysis

All statistical analyses were performed using IBM SPSS Statistics for Windows version 22.0 (IBM Corp., Armonk, NY, USA). Continuous variables were expressed as means ± standard deviations or medians with interquartile ranges (IQRs) or ranges (min–max), whereas categorical variables were summarized as frequencies and percentages. Differences between groups were assessed using the chi-square test or Fisher’s exact test for categorical variables and the Student’s t-test or Mann–Whitney U test for continuous variables, depending on the distribution of the data. Logistic regression analysis was performed to identify independent predictors of systemic reactions. Statistical significance was defined as a two-sided p < 0.05.

## 3. Results

A total of 623 pediatric patients with moderate-to-severe allergic rhinitis, with or without asthma, who underwent SCIT were included in the study: 325 patients from Hacettepe University and 298 patients from Dr. Behcet Uz Pediatric Diseases and Surgery Training and Research Hospital. Of these patients, 222 (35.6%) were female. The median age at immunotherapy initiation was 11.3 years (IQR, 9.0–13.9 years). Concomitant asthma was detected in 347 patients (55.7%). A total of 436 grass pollen SCIT courses and 207 house dust mite SCIT courses were administered, representing 643 distinct immunotherapy courses (Table 1).

**Figure 1:**
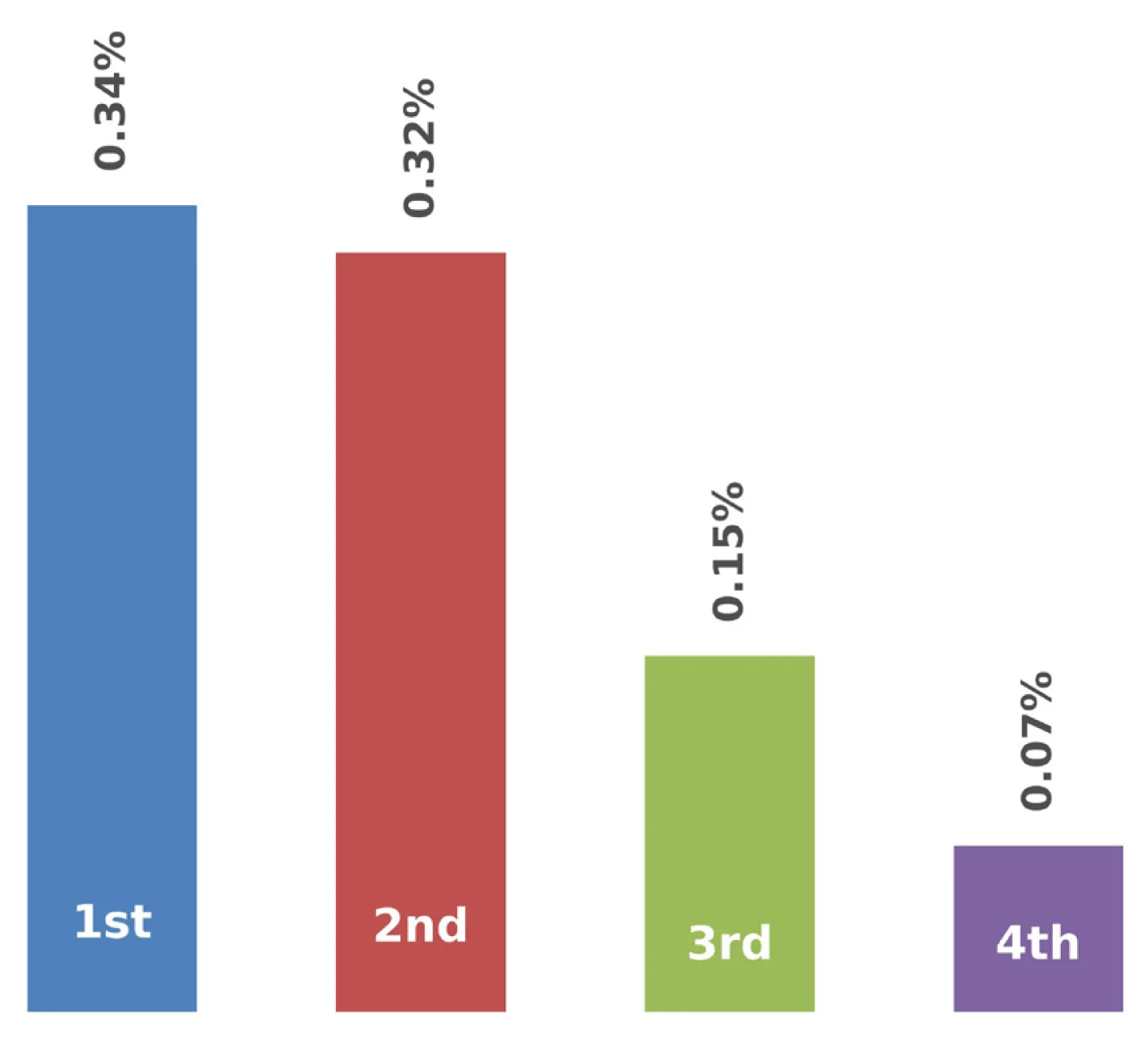
Annual incidence of systemic reactions during SCIT.

A total of 33,609 injections were administered to 623 patients included in the study. Systemic reactions occurred in 69 of 643 SCIT courses (10.7%), comprising 91 episodes, and resulting in an overall incidence of 0.27% per injection. Year-specific analyses revealed incidences of 0.34%, 0.32%, and 0.15% during the 1st, 2nd, and 3rd years of treatment, respectively (Figure 1). Analysis of treatment-related variables showed that systemic reactions occurred more frequently during the maintenance phase. Of the cases with systemic reactions, 14.5% occurred during the build-up phase, 76.8% occurred during the maintenance phase, and the remaining 8.7% were recorded during both phases. In contrast, neither extract manufacturer nor cumulative injection number was significantly associated with the occurrence of systemic reactions.

**Figure 2:**
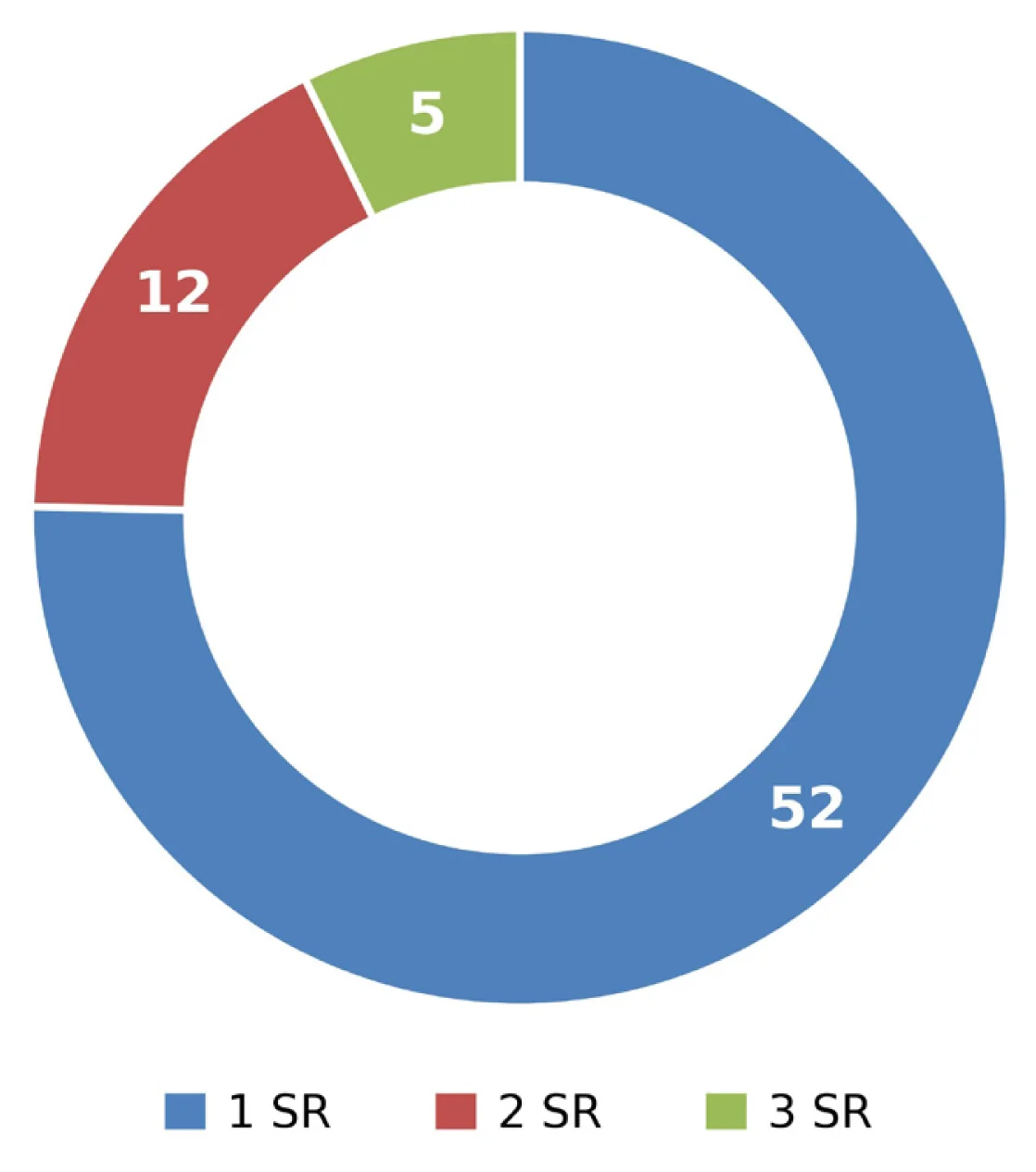
Distribution of systemic reaction episodes per patient.

Among patients who experienced SRs, five children developed three episodes each, 12 experienced two episodes, and 52 had a single episode during the course of immunotherapy (Figure 2).

**Figure 3:**
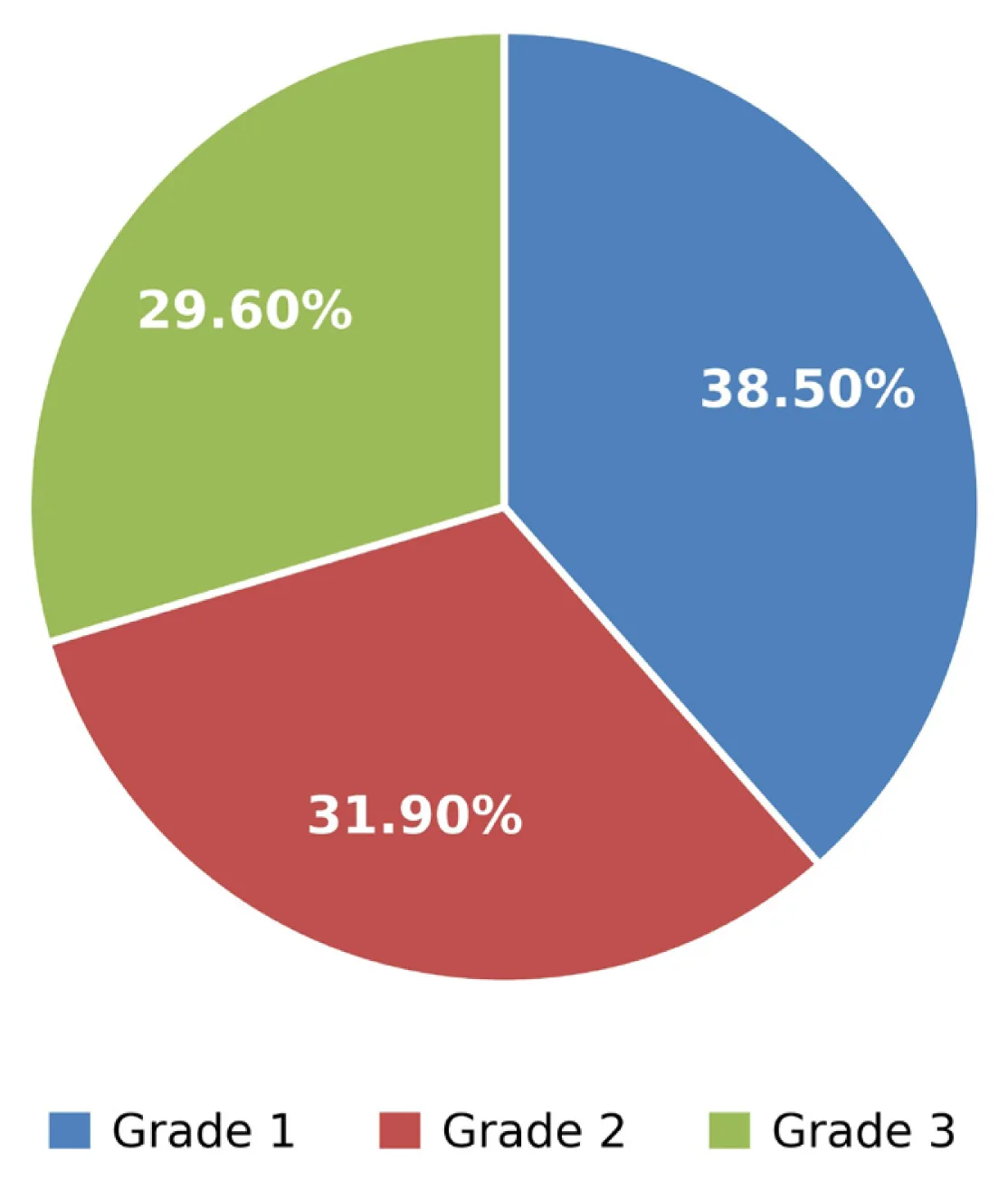
Severity distribution of systemic reactions according to the WAO 2024 sysytemic reactions grading system.

According to the WAO 2024 grading system, the severity of reactions was distributed as follows: 35 reactions (38.5%) were classified as grade 1, 29 reactions (31.9%) as grade 2, and 27 reactions (29.6%) as grade 3 (Figure 3).

No statistically significant difference was observed in the severity distribution between initial and recurrent SRs (p > 0.05). Dose adjustment and symptomatic treatment were implemented for grade 1–2 SRs, whereas immunotherapy was discontinued following grade 3 SRs.

Comparisons between patients with and without SR revealed no significant differences in demographic variables or baseline laboratory findings (Table 2). Logistic regression analysis did not identify significant predictors of SRs in the overall cohort or in the subgroup receiving house dust mite SCIT. In contrast, the presence of asthma in patients receiving grass pollen SCIT was associated with a significantly increased risk of SRs (OR, 2.345; 95% CI 1.128–4.877; p = 0.022) (Table 3). Moreover, comparisons between patients with and without recurrent SRs revealed similar age distributions, sex distributions, and laboratory profiles, whereas recurrence occurred at a significantly higher rate in the pollen SCIT subgroup (31.2% versus 9.5%; p = 0.047).

## 4. Discussion

In this study, SCIT was generally well tolerated in children with moderate to severe allergic rhinitis, with or without asthma, and systemic reactions remained infrequent despite the large number of administered injections. Most reactions occurred during the maintenance phase and were mild to moderate in severity, while only a small proportion of patients experienced recurrent episodes. Baseline demographic and laboratory characteristics did not differ significantly between patients with and without systemic reactions, suggesting that these variables were not predictive of systemic reactions in our cohort. However, the presence of asthma emerged as a notable risk factor among children receiving grass pollen immunotherapy and was associated with a higher likelihood of systemic reactions. Recurrent reactions were also observed more often in those undergoing pollen SCIT compared with house dust mite SCIT. Overall, these findings support the favorable safety profile of pediatric SCIT while suggesting that comorbid asthma and allergen type may influence individual risk. The frequency of SRs associated with SCIT reported in the literature has varied widely, with patient-based rates ranging from 0.06% to 3.2% and per-injection incidences of approximately 0.1%–0.2% [11–13]. In our cohort, the per-injection SR rate of 0.27% falls within the range reported in pediatric SCIT studies, although direct comparisons should be interpreted cautiously because published rates vary substantially according to allergen type, patient selection, extract formulation, treatment protocols, and adverse event reporting practices. International studies using standardized inhalant extracts have also confirmed the overall safety of SCIT, with systemic reaction rates reported within a similar range [14,15]. Similar variability has also been documented in a recent Turkish study evaluating subcutaneous immunotherapy with house dust mite extract in children with allergic rhinitis and/or asthma, which reported a per-injection systemic reaction rate of approximately 0.5% and identified no significant demographic or laboratory predictors of SRs [16]. In that study, systemic reactions occurred in a minority of patients and no severe or life-threatening events were reported, further supporting the general safety of pediatric SCIT. Taken together, data from our cohort and previous studies illustrate the broad range of SR rates across different settings, reflecting heterogeneity in allergen type (house dust mite [HDM] versus pollen), patient characteristics (monosensitized versus polysensitized, disease severity), and protocol-related factors (extract concentration, build-up strategy, and reporting standards). This variability reinforces the need to interpret SR rates within the context of clinical and methodological differences rather than as direct measures of treatment safety. The SR frequency observed in our cohort may partly reflect the tertiary referral nature of the participating centers, where patients with more complex or severe allergic diseases are more likely to be managed. However, this interpretation should remain cautious given the retrospective design and the heterogeneity of SCIT practices across studies.

Our data are also broadly consistent with a recent multicenter study from China that evaluated 3132 patients receiving 113,431 house dust mite SCIT injections between 2015 and 2023 and reported an SR rate of 9.6%, corresponding to 0.35% per injection [17]. In that study, identified risk factors included asthma, polysensitization, extract formulation and standardization, dosing errors, administration during acute exacerbations, injections from a newly prepared vial, and accelerated build-up protocols. In contrast, the only significant predictor identified in our cohort was asthma in the pollen SCIT subgroup. The identification of asthma as a risk factor for SRs in children receiving pollen SCIT is consistent with previous evidence linking uncontrolled asthma to increased risk during immunotherapy [3,6]. Although no significant predictors were found in the overall cohort or in the HDM subgroup, this finding still supports the need for careful assessment and close monitoring of asthmatic children undergoing SCIT.

Differences in allergen type, extract formulation, and regional clinical practices may account for the considerable inconsistency in risk factors reported in the literature. Some studies have identified biomarkers such as eosinophil counts and allergen-specific IgE levels as potential predictors of SRs. In contrast, other studies, including the recent Turkish HDM-SCIT study and Chinese preschool HDM-SCIT cohorts, found no demographic, laboratory, or clinical parameters that reliably predicted SRs [16,18,19]. Collectively, these discrepancies suggest that risk factors may be context-dependent, influenced by the allergen used, patient population, and protocol characteristics, and therefore may not be universally applicable. The fact that our study identified few predictive risk factors—apart from asthma in the pollen SCIT subgroup—is not surprising, given that other studies have also struggled to consistently identify predictors; however, this finding suggests that risk stratification remains imperfect. An important observation from our cohort is that SRs occurred more frequently during the maintenance phase, with more than three-quarters of reactions recorded during this period. Although previous reports have often emphasized the build-up phase as a higher-risk period, particularly when high-concentration vials or dose escalation are involved, our findings indicate that vigilance should be maintained throughout the full course of immunotherapy [20]. This pattern may partly reflect our clinical practice of avoiding SCIT initiation during the natural pollen season, which could reduce the likelihood of reactions during the build-up phase, especially in pollen-treated patients. Our observations are consistent with recent data from Xu et al., who similarly reported SRs distributed across both build-up and maintenance phases, supporting the view that systemic reactions are not restricted to periods of dose escalation [17]. Other pediatric and mixed-age studies also support the view that systemic reactions are not confined to the build-up phase. For example, a 2022 study of children receiving mite and pollen SCIT reported SRs during both treatment phases despite an overall low per-injection rate and identified local reactions as a factor associated with subsequent systemic reactions [21]. Likewise, a large Turkish series concluded that SCIT is generally safe but noted that local reactions were far more common than systemic ones, highlighting the need for routine monitoring even in children who initially demonstrate only local reactivity [22]. The finding that many SRs occur during the maintenance phase aligns with emerging evidence from mixed-age and pediatric cohorts indicating that the risk does not disappear after the build-up phase. In our cohort, the per-injection incidence of SRs declined over time, from 0.34% in the 1st year to 0.32% in the 2nd year and 0.15% in the 3rd year. Prior practice-based and review data indicate that SRs occur more frequently during build-up—particularly with accelerated protocols—and less commonly once patients are on steady maintenance schedules [20–24]. Our observation of a year-on-year reduction likely reflects (i) the transition from dose escalation to stable maintenance dosing, (ii) systematic dose adjustments following early reactions, and (iii) survivor-selection effects, whereby patients with poor tolerability discontinued treatment earlier. Notably, large real-life series similarly document that SRs are concentrated in phases of higher dosing intensity and diminish with sustained, well-controlled maintenance therapy, reinforcing the temporal decline we observed [5]. We also observed that recurrent SRs were more common in patients treated with pollen extracts, despite comparable demographic and laboratory profiles between recurrent and nonrecurrent cases. This observation aligns with the findings of Asero, who demonstrated that grass and ragweed pollen allergens represent significant independent risk factors for recurrent SRs during SCIT [25]. Together, these findings suggest that allergen type may influence not only the occurrence but also the recurrence of SRs. Collectively, our findings support the favorable safety profile of SCIT in children, with systemic reactions occurring infrequently and no fatal events observed. The slightly higher incidence observed in our cohort compared with some earlier reports emphasizes the importance of standardized documentation and multicenter surveillance for accurate risk estimation. Clinically, our findings support meticulous assessment of asthma status in children undergoing pollen SCIT and underscore the importance of maintaining vigilance beyond the build-up phase. Given the heterogeneity across studies, conclusions regarding safety should remain cautious and contextual. Although SCIT is generally well tolerated, the risk of SRs, including grade ≥3 reactions, remains; therefore, careful patient selection, monitoring, and informed consent are essential. The use of the updated WAO 2024 systemic reaction grading system also strengthens comparability with future studies and provides a more standardized framework for safety reporting.

The major strengths of this study include its relatively large pediatric cohort, its multicenter design, and the systematic application of the updated WAO 2024 systemic reaction grading system. However, several limitations should be acknowledged. The retrospective design may have resulted in the underestimation of mild or delayed events, and the inclusion of only two centers in Türkiye may limit the generalizability of the findings. Additionally, standardized data on asthma control were not consistently available, limiting interpretation of the association between asthma and SRs in the pollen SCIT subgroup. Although some treatment-related variables, including extract manufacturer, treatment phase, and cumulative injection number, were analyzed, other episode-specific factors such as vial changes, dose modifications, premedication, and preceding large local reactions were not consistently documented. Future prospective studies with broader geographic representation, consistent definitions, and standardized asthma control measurements will be essential for refining risk stratification in pediatric immunotherapy.

In conclusion, while the overall frequency of systemic reactions during pediatric SCIT was slightly higher than that reported in many previous studies, no fatal events were observed. Asthma was associated with an increased risk of SRs in the pollen SCIT subgroup, emphasizing the need for continued vigilance throughout the treatment course, including the maintenance phase. Taken together, these findings provide additional real-world evidence supporting the overall safety of SCIT in children.

## Figures and Tables

**Table 1:** Clinical and laboratory characteristics of the study population.

Patients’ demographic data	Total (n = 623)
**SCIT initial age median (IQR) (year**)	11.3 (9–13.9)
**Sex, n (%)**	
• **Female**	222 (35.6)
• **Male**	401 (64.4)
**Presence of asthma (n/%)**	347 (55.7)
**Total IgE (kU/L) median (IQR)**	257 (120–511)
**Eosinophil count (/mm** **^3^** **)**	300 (180–500)
**Eosinophil percent (%)**	3.9 (2.3–6.2)
**SCIT, n (%)**	
• **Grass pollen**	416 (66.7)
• **HDM**	187 (30)
• **Grass pollen + HDM**	20 (3.2)
**SCIT brand, n (%)**	
• **Allergopharma**	293 (45.6)
• **Novo-Helisen**	186 (28.9)
• **ALK**	156 (24.3)
• **Novo-Helisen + Allergopharma**	6 (0.9)
• **Phostal**	2 (0.3)

**Abbreviations:** HDM: house dust mite; IgE: immunoglobulin E; SCIT: subcutaneous allergen-specific immunotherapy.

**Table 2:** Clinical and laboratory characteristics according to the presence of systemic reactions at the SCIT course level.

	SR (n = 69)	No SR (n = 574)	p
**Age (year), median (IQR)**	11 (9–13.8)	11.2 (9–13.9)	0.764
**Sex, n (%)**			0.924
• **Male**	45 (65.2)	371 (64.6)	
• **Female**	24 (34.7)	203 (35.6)	
**Presence of asthma, n (%)**	44 (63.7)	317 (55.2)	0.177
**SCIT, n (%)**			0.741
• **HDM**	21 (30.4)	186 (32.4)	
• **Grass pollen**	48 (69.6)	388 (67.6)	
**Total IgE, median (IQR)**	394.5 (101.7–921.2)	250 (122–480)	0.09
**Eosinophil count (/mm** **^3^** **)**	300 (140–430)	300 (200–500)	0.270
**Eosinophil percentage (%)**	3.3 (1.7–5.65)	4 (2.3–6.3)	0.185
**Total injection number**	48.8 ± 26.8	52.7 ± 21	0.169

**Abbreviations:** HDM: house dust mite; IgE: immunoglobulin E; SCIT: subcutaneous allergen-specific immunotherapy; SRs: systemic reactions.

**Table 3:** Risk factors for systemic reactions in children receiving grass pollen SCIT.

	Univariable analysis		Multivariable analysis	
	OR (95% CI)	p	OR (95% CI)	p
**Eosinophil count (/mm** **^3^** **)**	1 (0.999–1.001)	0.659	1 (0.999–1.001)	0.721
**Total IgE, median (IQR)**	1 (1–1.001)	0.219	1 (1–1.001)	0.362
**Presence of asthma, n (%)**	1.669 (0.909–3.064)	0.099	**2.345 (1.128**–**4.877)**	**0.022**

**Abbreviations:** IgE: immunoglobulin E; SCIT: subcutaneous allergen-specific immunotherapy.

## References

[1] Calderón MA Cox L Casale TB Moingeon P Demoly P Multiple-allergen and single-allergen immunotherapy strategies in polysensitized patients: Looking at the published evidence Journal of Allergy and Clinical Immunology 2012 129 4 929 934 10.1016/j.jaci.2011.11.019 22244595

[2] Durham SR Walker SM Varga EM Jacobson MR O’Brien F Long-term clinical efficacy of grass-pollen immunotherapy The New England Journal of Medicine 1999 341 7 468 475 10.1056/NEJM199908123410702 10441602

[3] Epstein TG Liss GM Berendts KM Bernstein DI AAAAI/ACAAI subcutaneous immunotherapy surveillance study (2013–2017): fatalities, infections, delayed reactions, and use of epinephrine autoinjectors The Journal of Allergy and Clinical Immunology: In Practice 2019 7 6 1996 2003e1 10.1016/j.jaip.2019.01.058 30776526

[4] Bernstein DI Epstein TEG Safety of allergen immunotherapy in North America from 2008–2017: Lessons learned from the ACAAI/AAAAI National Surveillance Study of adverse reactions to allergen immunotherapy Allergy and Asthma Proceedings 2020 41 2 108 111 10.2500/aap.2020.41.200001 32122446

[5] Robertson K Montazeri N Shelke U Jeimy S Kim H A single centre retrospective study of systemic reactions to subcutaneous immunotherapy Allergy, Asthma, and Clinical Immunology 2020 16 93 10.1186/s13223-020-00491-5 PMC7607692 33292437

[6] Lim CE Sison CP Ponda P Comparison of systemic reactions in children and adults receiving subcutaneous immunotherapy Journal of Allergy and Clinical Immunology: In Practice 2017 5 5 1241 1247e2 10.1016/j.jaip.2017.01.014 28341172

[7] Gur Cetinkaya P Kahveci M Esenboğa S Karaatmaca B Soyer O Systemic and large local reactions during subcutaneous grass pollen immunotherapy in children Pediatric Allergy and Immunology 2020 31 6 643 650 10.1111/pai.13261 32320504

[8] Venkatesan P 2025 GINA report for asthma The Lancet Respiratory Medicine 2025 S2213-2600(25)00242-5 10.1016/S2213-2600(25)00242-5 40582369

[9] Bousquet J Khaltaev N Cruz AA Denburg J Fokkens WJ Allergic Rhinitis and its Impact on Asthma (ARIA) 2008 update (in collaboration with the World Health Organization, GA(2)LEN and AllerGen) Allergy 2008 63 8 160 10.1111/j.1398-9995.2007.01620.x 18331513

[10] Turner PJ Ansotegui IJ Campbell DE Cardona V Carr S Updated grading system for systemic allergic reactions: Joint Statement of the World Allergy Organization Anaphylaxis Committee and Allergen Immunotherapy Committee World Allergy Organization Journal 2024 17 3 100876 10.1016/j.waojou.2024.100876 38361745 PMC10867340

[11] Can D Demir E Tanaç R Gülen F Yenigün A Immediate adverse reactions to immunotherapy Journal of Investigational Allergology and Clinical Immunology 2003 13 3 177 180 14635467

[12] Nacaroglu HT Erdem SB Sumer O Karaman S Ünsal Karkıner CS Local and systemic reactions to subcutaneous allergen immunotherapy: Ten years’ experience in a pediatric clinic Annals of Allergy, Asthma and Immunology 2016 116 4 349 353 10.1016/j.anai.2016.01.015 26905639

[13] Dursun AB Sin BA Oner F Misirligil Z The safety of allergen immunotherapy in Turkey Journal of Investigational Allergology and Clinical Immunology 2006 16 2 123 128 16689186

[14] Nettis E Giordano D Pannofino A Ferrannini A Tursi A Safety of inhalant allergen immunotherapy with mass units-standardized extracts Clinical and Experimental Allergy 2002 32 12 1745 1749 10.1046/j.1365-2222.2002.01544.x 12653166

[15] Arifhodzic N Behbehani N Duwaisan AR Al-Mosawi M Khan M Safety of subcutaneous specific immunotherapy with pollen allergen extracts for respiratory allergy International Archives of Allergy and Immunology 2003 132 3 258 262 10.1159/000074307 14646387

[16] Altas U Cetemen A Altas ZM Akkelle E Ozkars MY Retrospective evaluation of adverse reactions after subcutaneous allergen-specific immunotherapy in children with house dust mite allergy Northern Clinics of Istanbul 2023 10 5 675 680 10.14744/nci.2023.78871 37829749 PMC10565755

[17] Xu Q Jia J Lin H Liang D Chen H Systemic reactions to house dust mite subcutaneous immunotherapy in patients with allergic rhinitis and/or asthma: A real-life, multicenter study (2015–2023) Allergy 2025 80 5 1506 1508 10.1111/all.16254 39056450 PMC12105055

[18] Wang X Wang L Zheng X Xiao X Li L Mo L Analysis of systemic reactions during house dust mite subcutaneous immunotherapy in children with allergic rhinitis Journal of New Medicine 2024 55 7 534 540

[19] Yang Y Ma D Huang N Li W Jiang Q Safety of house dust mite subcutaneous immunotherapy in preschool children with respiratory allergic diseases Italian Journal of Pediatrics 2021 47 1 101 10.1186/s13052-021-01046-z 33892756 PMC8063484

[20] Aue A Ho J Zhu R Kim H Jeimy S Systemic reactions to subcutaneous allergen immunotherapy: real-world cause and effect modelling Allergy, Asthma, and Clinical Immunology 2021 17 1 65 10.1186/s13223-021-00566-x PMC8262003 34229743

[21] Duman Senol H Topyildiz E Ekici B Gulen F Demir E Effectiveness and adverse reactions to subcutaneous immunotherapy in children with allergic rhinitis/asthma International Journal of Pediatric Otorhinolaryngology 2022 162 111292 10.1016/j.ijporl.2022.111292 36007303

[22] Toprak-Kanık E Yılmaz Ö Yangın-Ergon E Türkeli A Yüksel H Safety of subcutaneous allergen immunotherapy in children: A retrospective review and bird eye to literature The Turkish Journal of Pediatrics 2018 60 6 684 690 10.24953/turkjped.2018.06.009 31365205

[23] Gomes Dos Reis Pimentel RA Oliveira G Ferreira Chaves Loureiro E Lemos CS Accelerated subcutaneous immunotherapy in pediatric population - Systematic review Pulmonology 2018 24 3 182 193 10.1016/j.rppnen.2017.07.009 29183774

[24] Liu JL Ning WX Li SX Xu YC Wu L The safety profile of subcutaneous allergen immunotherapy in children with asthma in Hangzhou, East China Allergologia et Immunopathologia 2017 45 6 541 548 10.1016/j.aller.2017.04.002 28629672

[25] Asero R Detection of risk factors for systemic adverse reactions to SCIT with natural depot allergen extracts: a retrospective study European Annals of Allergy and Clinical Immunology 2015 47 6 211 217 26549339

